# Metabolic syndrome in childhood cancer survivors

**DOI:** 10.17179/excli2021-3916

**Published:** 2022-02-10

**Authors:** Amena Firoz, Muhammad Haris

**Affiliations:** 1 Pondicherry Institute of Medical Sciences, Puducherry, India; 2 University Hospital Morecambe Bay Trust, Lancaster, United Kingdom

## ⁯

Every 3 minutes a parent is heartbroken by their child's cancer diagnosis (ACCO, 2021[1]) Cancer is considered the second leading cause of mortality in children under 15 years of age and it has been predicted that in 2021 around 10,500 children in this age group will be diagnosed with cancer (ACS, 2021[2]). Leukemia (29 %), central nervous system tumors (26 %), and lymphoma-Hodgkin and Non-Hodgkin (10 %) are the most common childhood malignancies (ASCO, 2021[4]).

Over the last few decades, cutting-edge advances in cancer treatment have raised the 5-year cancer survival rate from 58 % in the mid-1970s to 84 % in 2019 (ACS, 2021[2]). On one hand, these advancements have given many families a ray of hope but on the other, they have made survivors vulnerable to the various treatment-related side effects thereby gradually increasing morbidity and mortality.

Traditional cancer treatments like radiotherapy, chemotherapy, glucocorticoid therapy, and surgery provide a conducive medium for various vascular and metabolic alterations which lead to many long-term sequelae (Casco and Soto-Vega, 2016[9]). These treatments combined with ultra-modern cancer remedies like immunotherapies have deep-rooted effects on a child's growing metabolic system. These potential effects of these therapies on metabolic genetics at a molecular level are often underappreciated, overlooked, and not well understood given the scarcity of data (Pluimakers et al., 2019[24]). There is a lack of understanding of the effects of a myriad of cancer therapies on metabolism and inadequate predictive biomarkers for endo-hormonal disease come forth as a significant challenge for pediatric age groups (Brignardella et al., 2013[8]).

Endocrinopathies like diabetes mellitus (hereafter abbreviated as diabetes) and metabolic syndrome are stated as the most common treatment-related complication with around 50 % of survivors having at least one hormonal disturbance (Brignardella et al., 2013[8]). Cardiovascular disease is known to be the second common cause of mortality in childhood cancer survivors with the prime risk factor being metabolic syndrome and its individual components (Armstrong et al., 2016[3]).

Metabolic syndrome is a constellation of metabolic abnormalities including insulin resistance, systemic hypertension, central obesity, and dyslipidemia (Rochlani et al., 2017[25]). The pathogenesis of metabolic syndrome includes both genetic factors and acquired factors like insulin resistance, neurohormonal activation, and chronic inflammation that leads to cardiovascular diseases (Rochlani et al., 2017[25]). Abnormal signaling mechanisms in adipose tissue including enhanced inflammatory response and endothelial damage are described as key mechanisms for the development of metabolic syndrome (Casco and Soto-Vega, 2016[9]). This combined with direct damage to the target organ system by radiation therapy and chemotherapeutic damage to endocrine signaling and second messenger system is argued to be contrivanced to the origination of metabolic syndrome (Casco and Soto-Vega, 2016[9]). Figure 1 (supplementary information) is a graphical representation of the clinical diagnosis of metabolic syndrome. 

This letter aims to illustrate the pathophysiology of metabolic syndrome in childhood cancer survivors, learn about the different methods of screening, and early detection of metabolic syndrome, and also focus on the prevention strategies in order to increase the survival of childhood cancers. 

Metabolic syndrome as mentioned earlier is a cluster of symptoms, the pathogenesis of which is not completely known. Visceral adiposity has been shown to be the primary start point leading to the development of metabolic syndrome (Rochlani et al., 2017[25]). The three main proposed pathways are insulin resistance, neurohormonal activation, and chronic inflammation (Rochlani et al., 2017[25]). Insulin plays a key role in glucose uptake by muscle and liver, hepatic gluconeogenesis, and inhibiting lipolysis hence insulin resistance nullifies these effects leading to an increase in the amount of circulating free fatty acids (Boden and Shulman, 2002[7]). Adipocytes are demonstrated to have endocrine and immune properties, thus producing adipokines like leptin and adiponectin, and angiotensin II (Ang II) which are vital in the neurohormonal pathway (Rochlani et al., 2017[25]). Adiponectin is a protective adipokine that is anti-inflammatory and anti-atherogenic and is decreased when adipose tissue mass increases. Reactive oxygen species production via Ang II activation also contributes to metabolic syndrome and cardiovascular disease as they cause endothelial damage, platelet aggregation, and oxidation of lipids (Mehta et al.,2007[23]). Lastly, the final pathway is regulated by inflammatory markers like tumor necrosis factor-alpha (TNF alpha), interleukin 6, and C reactive protein (CRP) which cause lipolysis and increase prothrombotic state, thereby leading to metabolic syndrome and cardiovascular diseases (Rochlani et al., 2017[25]). Many studies have shown the incidence of metabolic syndrome and its components in Childhood Cancer Survivors. In recent times this trend has increased and Figure 2 (supplementary information) highlights the risk factor attributed to the development of metabolic syndrome.

## Pathophysiology of the Components of Metabolic Syndrome in Childhood Cancer Survivors

### Diabetes mellitus

Diabetes is described as hyperglycemia caused by either insufficient insulin secretion due to autoimmune destruction of pancreatic β cells or insulin resistance. In recent times it has been shown that the development of diabetes has increased in childhood survivors, especially if treated at a young age or exposed to abdominal or total body irradiation (Friedman et al., 2019[17], 2020[16]). Abdominal radiation is the most important form of therapy for a variety of solid malignancies like neuroblastoma and Wilms tumors. It has been studied that survivors of neuroblastoma and Wilms tumors treated with abdominal radiation had a 6.9-fold and 2.1-fold increased risk of diabetes respectively as compared to their siblings (Meacham et al., 2009[22]). The underlying mechanism is unclear but being attributed to pancreatic insufficiency caused due to destruction of the tail of the pancreas by radiation (de Vathaire et al., 2012[12]). Total body irradiation is a prerequisite for children undergoing hematopoietic cell transplantation (HCT) for hematologic malignancies which is very common in children. It has been shown that survivors treated with total body irradiation have a 12.6 fold increase in risk for developing diabetes as compared to their siblings (Meacham et al., 2009[22]). This has also been shown in a study conducted by Bielorai et al. (2018[6]) where they also state central obesity along with age, diet, physical activity as risk factors. The main underlying mechanism is thought to be the growth of insulin resistance but some studies also attribute this to hindrance in the hypothalamic-pituitary axis leading to endocrinopathies causing growth hormone deficiency (Taskinen et al., 2007[27]). The use of an exogenous steroid for hematologic malignancies like Acute Lymphoblastic Leukemia (ALL) has also shown to cause temporary glucose impairments which can persist even after cessation of therapy (Friedman et al., 2019[17]; Banihashem et al., 2014[5]). William et al. (2020[28]) in their study concluded that adult survivors of ALL who developed diabetes during treatment and required regular monitoring as they are prone to develop diabetes later on in life. A study conducted by Winther et al. (2018[29]) shows that childhood cancer survivors with diabetes have a greater risk of developing cardiovascular disease than survivors without diabetes. Hence individuals exposed to abdominal radiation or total body irradiation should be periodically followed up with fasting blood sugar or hemoglobin A1c and more closely if there is any family history of diabetes mellitus (COG, 2018[11]). Table 1 (supplementary information) summarizes the studies we reviewed from which we learnt about diabetes in Childhood Cancer Survivors.

### Hypertension

Hypertension is a consequence of arteriolar vasoconstriction leading to blood exerting extra force against the vessels resulting in heart strain. Therefore, hypertension is considered one of the greatest cardiovascular determinants for morbidity and mortality. St. Jude lifetime cohort study (SJLIFE) concluded that more than one third were found to have pre-hypertension, thus increasing lifetime risk for hypertension (Gibson et al., 2017[19]). However, Gibson et al. opined that it is likely that development of hypertension is due to multifactorial causation and thus needs enhanced clinical attention (Gibson et al., 2017[19]).

Similarly, England et al. provided the exon sequence of the genes determinant of cardiometabolic complications in childhood Acute Lymphoblastic Leukemia (cALL) (England et al., 2017[14]). Variants in genes BCL-2 associated agonist of cell death (BAD) and Fc-R related receptor-3 (FCRL-3) are associated with extreme cardiometabolic complications (England et al., 2017[14]). Interestingly, these markers can be used as a predictor for metabolic complications. A common variant *rs2286615* in BAD gene is linked with obesity, insulin resistance and dyslipidemias (England et al., 2017[14]). Likewise, corticotropin releasing hormone receptors (CRH 1 & 2) were linked to the development of pre-hypertension (England et al., 2017[14]). Moreover, CRH also uncovered a gender related phenotype, with men being affected with hypertension more compared to women. Overall, this study provided great insights into the genetic determinants and ways in which we can slow the risk of development of cardiometabolic complications (England et al., 2017[14]).

### Dyslipidemia and obesity

A large number of children with cancers are treated with corticosteroids and cranial irradiation. There has been a significant increase in net body fat has been reported with patients receiving steroids (Caubet Fernandez et al., 2019[10]). All the adipose parameters including lean body mass, total body weight and serum leptin levels were affected (Caubet Fernandez et al., 2019[10]). The net result of pulsed steroids were increased fat content and leptin levels and reduced lean body mass (Caubet Fernandez et al., 2019[10]). This effect is noted to be amplified when platinum based agents were combined with steroids (Caubet Fernandez et al., 2019[10]).

Innumberale chemotherapeutic agents have been linked with an enhanced inflammatory reaction in the adipose tissue, thus leading to obesity. This causal relationship has been extensively explored by Caubet Fernandez et al. (2019[10]), who opined through a Bayesian model that anticancer antibiotic Doxorubicin has been extensively linked with insulin resistance through its effect on inflammatory reaction in the fat cells. 

Lupo et al. (2019[21]) studied the link between BMI-DNA methylation loci and obesity among the childhood ALL survivors. They concluded that in populations of ALL survivors who received chemotherapy, BMI-DNA methylation loci is more strongly linked to obesity compared to the normal population.

Foster et al. (2019[15]) have concluded in their multi-ethnic cohort that various novel chemotherapies for targeting ALL have been linked to weight gain. Further interpretation from this study highlights the fact that weight gain is at its pinnacle one month post-treatment and one year weight post-treatment is often maintained at five year post-treatment (Foster et al., 2019[15]). Compellingly enough, these numbers provide significant breakthroughs making monitoring and early intervention possible.

Furthermore, Lorenc et al. (2020[20]) systematically analyzed body compositions in cancer survivors following hematopoietic stem cell transplant and total body irradiation (HSCT and TBI). They deduced that post HSCT and TBI, some patients are likely to have increased fat redistribution in the form of central obesity and altered fat tissue function. Moreover, muscle mass was significantly lower compared to a healthy population with impaired muscle function (Lorenc et al., 2020[20]).

Sims et al. (2020[26]) identified leptin as a key biomarker for obesity in childhood brain tumor survivors. Among these cancers, craniopharyngioma has been linked with increased leptin levels thus causing hypothalamic obesity. Table 2 (supplementary information) is a tabular representation of the predictive biomarkers for cardiometabolic complications. In addition Table 3 (supplementary information) summarizes the studies we reviewed from which we learnt about hypertension and dyslipidemia in childhood cancer survivors.

### Prevention

All childhood cancer survivors should be counseled annually on the importance of regular physical activity and a heart-healthy diet. 

In order to increase the free and overall cancer survival, weight management program and physical activity have been linked with beneficial outcomes (Demark-Wahnefried et al., 2018[13]). However, further studies are needed as to how these interventions affect at population level (Demark-Wahnefried et al., 2018[13]).

Zhang et al. (2019[30]) like Foster et al. (2019[15]) opined that in ALL the most sensitive window for weight gain is immediate post treatment and any intervention in this critical time period can be instrumental. Besides that, they also ventured that early dietary intercessions which include high protein, good carbohydrates and utilization of milk have been preliminary linked with benefit (Zhang et al., 2019[30]).

Although existing guidelines can be utilized for primary prevention of cardiometabolic complications, Gibson et al. (2016[18]) calls for a modified strategy and an individual based approach to minimize the development of cardiometabolic complications.

### Limitations

We searched three databases which highlight the fact that we might have missed some papers. It was noticed that since it takes decades for cardiometabolic complications, many studies did not follow the subjects for a longer period of time to prove the causal link. Metabolic syndrome presents as diverse disease in different ethnicities thus an in-depth analysis of confounding factors is required. Various genetic biomarkers have been identified to be early predictors for cardiometabolic complications, however dearth of evidence on prevention of these complications calls for large scale studies.

### Conclusion

We focused on the association of metabolic syndrome in childhood cancer survivors as this has become a concern in recent times. Treatment advances and other risk factors have increased the prevalence of metabolic syndrome and thereby cardiovascular disease in these survivors. 

Treatments like total body irradiation and exogenous steroids have been shown to promote insulin resistance, weight gain and inflammatory response in the childhood cancer survivors.

Additionally, chemotherapeutic agents have been demonstrated to enhance the adipose tissue inflammation and alter weight parameters. In addition, several novel predictive biomarkers have been collected, indicating early diagnosis and intervention. 

In conclusion, preventive mechanisms such as regular monitoring of HbA1c, blood pressure, and lipid profile are very important in survivors of childhood cancer through adulthood. Educating families and spreading awareness about the long-term effects of treatments with enhanced follow-up of these patients is pivotal in preventing cardiovascular morbidity in the pediatric cancer population. 

However, our analysis calls for further studies to demonstrate how particular treatment modification can help reduce risk of cardiometabolic complications. How a particular biomarker can predict long-term complications, the timing of intervention, what factors affect the outcomes of particular treatments, and when and how long to follow-up require further large-scale and comprehensive studies. 

We also hope that our survey will motivate future researchers to discover new treatment strategies, especially for childhood malignancies which have less long-term side effects on growing children.

## Supplementary Material

Supplementary information
