# Supplementary material for: Metabolic syndrome in childhood cancer survivors
Source: EXCLI J. 2022 Feb 10;21:380–6. doi: 10.17179/excli2021-3916 (PMC8971347; doi:10.17179/excli2021-3916)
Supplement: Supplementary information [file EXCLI-21-380-s-001.pdf]

EXCLI Journal 2022;21:380-386 – ISSN 1611-2156
Received: May 25, 2021, accepted: February 09, 2022, published: February 10, 2022

Supplementary information to:

Letter to the editor:

METABOLIC SYNDROME IN CHILDHOOD CANCER SURVIVORS

Amena Firoz[1*], Muhammad Haris[2]

[1] Pondicherry Institute of Medical Sciences, Puducherry, India
[2] University Hospital Morecambe Bay Trust, Lancaster, United Kingdom

* **Corresponding author:** Amena Firoz, Pondicherry Institute of Medical Sciences, Puducherry, India, E-mail: amenafiroz02@gmail.com

https://dx.doi.org/10.17179/excli2021-3916

This is an Open Access article distributed under the terms of the Creative Commons Attribution License (http://creativecommons.org/licenses/by/4.0/).

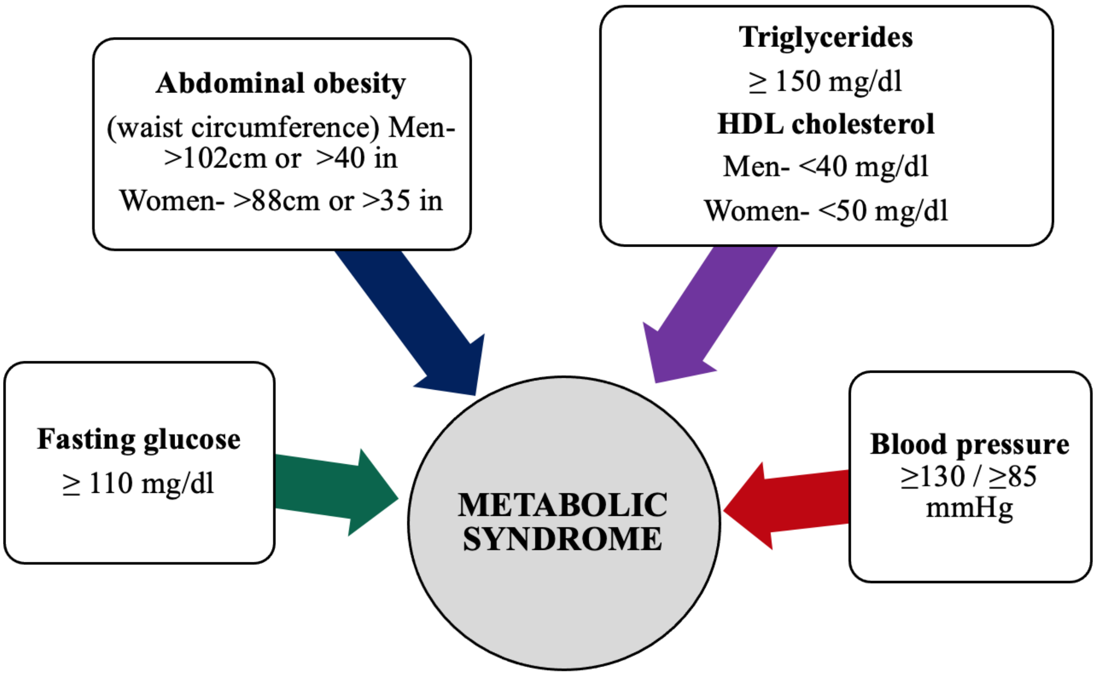

Figure 1: Clinical diagnosis of metabolic syndrome

EXCLI Journal 2022;21:380-386 – ISSN 1611-2156
Received: May 25, 2021, accepted: February 09, 2022, published: February 10, 2022

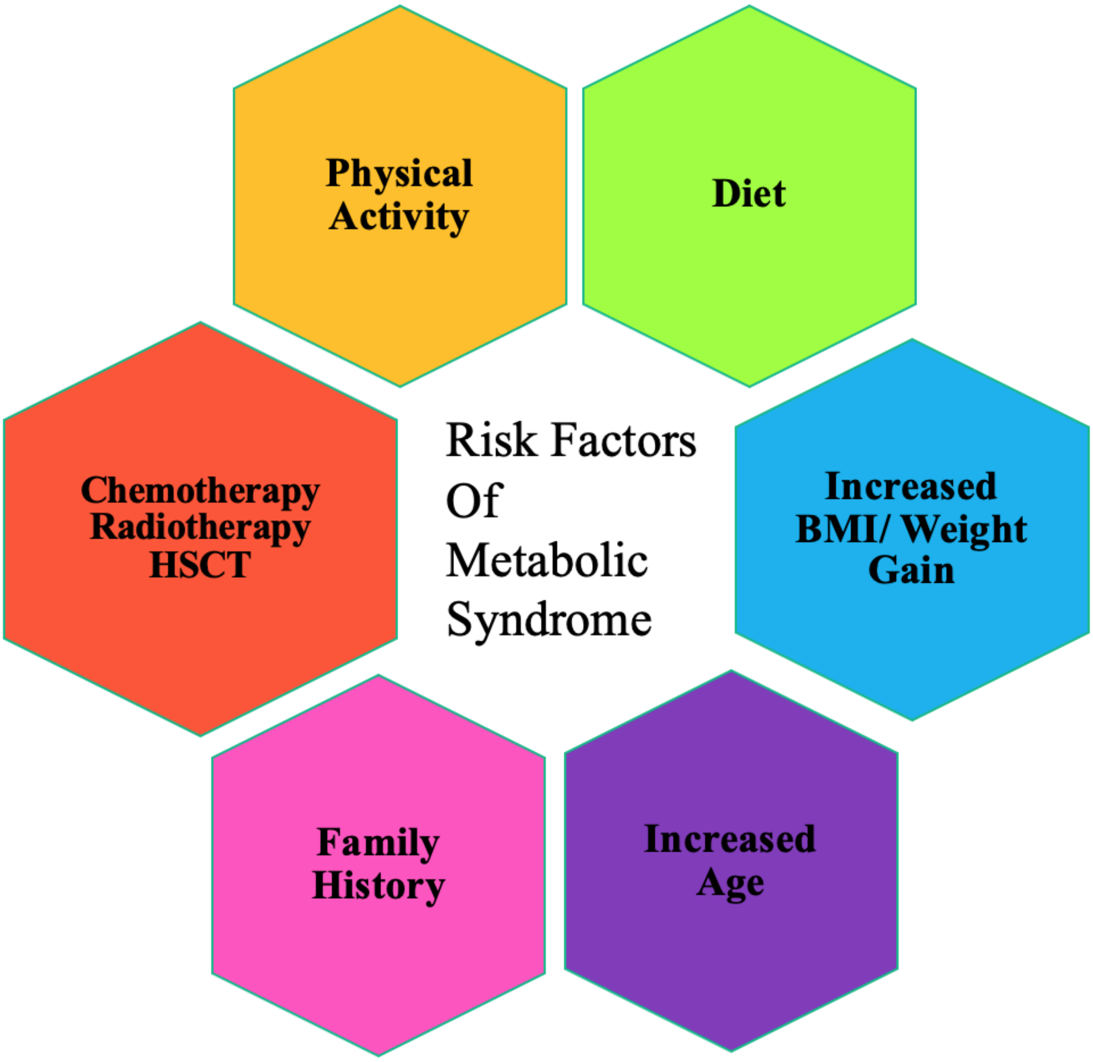

Figure 2: Risk factors for metabolic syndrome

EXCLI Journal 2022;21:380-386 – ISSN 1611-2156
Received: May 25, 2021, accepted: February 09, 2022, published: February 10, 2022

Table 1: Studies which discuss diabetes in childhood cancer survivors

Author Name	Year	Type of study	Results and conclusions
Friedman et al.	2019	Traditional review	Cranial, abdominal, and total body irradiation and glucocorticoids have been identified to cause diabetes in cancer survivors but the underlying mechanism is uncertain.
Friedman et al.	2020	Observational study	Among survivors treated with abdominal radiation, the risk of developing diabetes was higher in young age patients with higher BMI and higher pancreatic tail dose.
Bielorai et al.	2018	Traditional review	Pediatric patients who undergo HSCT and total body irradiation have increased chances of developing Type 2 diabetes compared to ALL survivors. The main risk factor is said to be central obesity along with age, diet, physical activity.
Williams et al.	2020	Observational study	Increased age and BMI along with a history of development of diabetes during treatment make adult survivors of childhood ALL susceptible to diabetes mellitus later in life and hence need to be monitored.
Winther et al.	2018	Observational study	Childhood cancer survivors with diabetes have a higher risk of cardiovascular diseases than those without diabetes.

Table 2: Predictive biomarkers for cardiometabolic complications

Author	Year	Biomarker/Genes loci	Prediction
England et al.	2017	BAD and FCRL-3	Obesity and insulin resistance
Lupo et al.	2019	BMI-DNA methylation loci	Obesity
England et al.	2017	CRH (1 and 2)	Prehypertension
Sims et al.	2020	Leptin	Total and central obesity

EXCLI Journal 2022;21:380-386 – ISSN 1611-2156
Received: May 25, 2021, accepted: February 09, 2022, published: February 10, 2022

Table 3: Studies which talk about hypertension and dyslipedemia in childhood cancer survivors

Author Name	Year	Type of study	Results and conclusions
Gibson et al.	2017	Observational study	Hypertension prevalence was found to be 70 % in cancer survivors who are in the 5th decade of life without any specific treatment predilection. About a quarter of the patient involved in the study developed prehypertension.
England et al.	2017	Observational study	Two susceptible loci contributing to cardiometabolic complications were identified in this study. These gene identifiers can be utilized for the early detection of obesity, hypertension, and insulin resistance in cancer survivors.
Caubet Fernandez et al.	2019	Observational study	Although cranial irradiation and steroid treatment independently enhance the risk of pre-hypertension, abnormal lipid profile, and obesity, the combined risk of the two therapies was not linked with an enhanced likelihood of obesity and hypertension.
Lupo et al.	2019	Observational study	ALL therapy is linked with obesity with 39 loci identified with link to chemotherapy. However, cranial radiation for ALL is not syndicated with enhanced risk.
Foster et al.	2019	Observational study	Weight gain was found to be linked very strongly in the immediate year following therapy, thus this window can be targeted in lifestyle management.
Lorenc et al.	2020	Systematic review	A select few cases were identified to have increased in central obesity post ALL treatment, but muscle mass was found to be significantly lower.
Sims et al.	2020	Traditional review	Leptin is linked as a potential biomarker for central obesity in pediatric central nervous tumors.
Demark-Wahnefried et al.	2018	Traditional review	Lifestyle modifications including exercise and weight loss have been linked to reduced cardiovascular morbidity and mortality in cancer survivors.
Zhang et al.	2019	Original research	Various lifestyle interventions were accessed to reduce the risk of weight gain post-therapy in cancer survivors. This study indicates that early intervention in lifestyle is proven to be efficacious.
Gibson et al.	2016	Traditional review	Cites lack of high evidence data on management of cardiometabolic complications in childhood cancer survivors.

All references cited here can be found in the main document: https://dx.doi.org/10.17179/excli2021-3916.
